# Segregation but Not Replication of the *Pseudomonas aeruginosa* Chromosome Terminates at *Dif*

**DOI:** 10.1128/mBio.01088-18

**Published:** 2018-10-23

**Authors:** Bijit K. Bhowmik, April L. Clevenger, Hang Zhao, Valentin V. Rybenkov

**Affiliations:** a Department of Chemistry and Biochemistry, University of Oklahoma, Norman, Oklahoma, USA; Duke University School of Medicine

**Keywords:** DNA replication, FROS, GC-skew, *Pseudomonas aeruginosa*, chromosome segregation, fluorescence microscopy

## Abstract

Coordination between chromosome replication and segregation is essential for equal partitioning of genetic material between daughter cells. In bacteria, this is achieved through the proximity of the origin of replication, *oriC*, and the chromosome partitioning site, *parS*. We report here that in Pseudomonas aeruginosa, segregation but not replication is also controlled at the terminus region of the chromosome. Using the fluorescent repressor operator system (FROS), we investigated chromosome segregation in P. aeruginosa strain PAO1-UW, wherein the chromosome dimer resolution site, *dif*, is asymmetrically positioned relative to *oriC*. In these cells, segregation proceeded sequentially along the two chromosomal arms and terminated at *dif*. In contrast, chromosome replication terminated elsewhere, opposite from *oriC*. We further found two large domains on the longer arm of the chromosome, wherein DNA segregated simultaneously. Notably, GC-skew, which reflects a bias in nucleotide usage between the leading and lagging strands of the chromosome, switches polarity at the *dif* locus but not necessarily at the terminus of replication. These data demonstrate that termination of chromosome replication and segregation can be physically separated without adverse effects on bacterial fitness. They also reveal the critical role of the *dif* region in defining the global layout of the chromosome and the progression of chromosome segregation and suggest that chromosome packing adapts to its subcellular layout.

## Abstract (precis)

Segregation of genetic information is a central event in cellular life. In bacteria, chromosome segregation occurs concurrently with replication, sequentially along the two arms from *oriC* to *dif*. How the two processes are coordinated is unknown. We explored here chromosome segregation in an opportunistic human pathogen, Pseudomonas aeruginosa, using its strain with markedly unequal chromosomal arms. We found that replication and segregation diverge in this strain and terminate at very different locations, whereas the longer chromosomal arm folds into large domains to align itself with the shorter arm. The significance of this research is in establishing that segregation and replication of bacterial chromosomes are largely uncoupled from each other and that the large-scale structure of the chromosome adapts to its subcellular layout.

## INTRODUCTION

Chromosome replication and segregation are two central events in the life of a cell. Coordination between these two processes is essential for equal genome partitioning between daughter cells. Eukaryotic cells achieve this by limiting these two events to once per cell cycle, each at its own stage. Once replication is complete, attachment of a microtubule-based mitotic spindle via a kinetochore promotes sister chromosome segregation into daughter cells (1). In bacteria, these events occur concurrently where the duplication of chromosomal loci is quickly followed by their segregation (2–5).

The key event that establishes this coordination occurs shortly after the initiation of replication and is mediated by the ParAB*S* system. This system consists of a conserved DNA sequence, *parS*, the *parS*-binding protein ParB, and an actin-like protein, ParA (6–9). This system has been found in diverse bacteria, and bacteria with multiple chromosomes harbor unique *parS*-ParB pairs on each of them (10, 11). Invariably, *parS* is located close to the origin of replication and cannot be relocated far from it, suggesting a functional or structural interaction between chromosome replication and segregation (9, 12–14). Whether or not such an interaction exists past the initiation event remains unknown.

The terminus region of the chromosome contains another genetic element that controls chromosome dynamics. The *dif* site serves as the recognition sequence for XerCD, a site-specific recombinase, which ensures resolution of chromosome dimers prior to cell division. The assembly of the recombination complex is facilitated by a septum-tethered DNA translocase FtsK, which ensures the capture of the two recombining *dif*s in the vicinity of the septum and activates the complex for recombination (15, 16). About half of all presently sequenced eubacterial genomes contain a canonical *dif* site, and several alternative *dif*s were found in other bacteria (17). Translocation of FtsK toward *dif* is guided by numerous KOPS (FtsK orienting polar sequences), which are distributed along both arms from *oriC* to *dif* (18, 19). The sequence of KOPS, GGGNAGGG, is asymmetrically distributed in numerous bacteria (20).

In Escherichia coli and Bacillus subtilis, *dif* is flanked by several polar *Ter* sites. The binding of Tus (in E. coli) or RTP (in B. subtilis) protein to *Ter* creates a roadblock to forks advancing from the opposite arm, but not its own, thereby precluding chromosome overreplication (21, 22). This arrangement of the *Ter* and *dif* sites ensures a degree of coordination between chromosome replication and dimer resolution. In these bacteria, chromosome segregation proceeds along the two arms from *oriC* to *dif*. As a result, termination of replication, XerCD recombination, and chromosome segregation all occur at the same chromosomal locus. Notably, the activity of *dif* is not affected by relocation of the terminus of replication, suggesting that the two processes are not biochemically coordinated (23).

Bacterial chromosomes display a nucleotide usage bias between the leading and lagging strands. In general, the leading strand has an excess of guanines, while the lagging strand contains an excess of cytosines. The resulting GC-skew changes the polarity in the vicinity of *oriC* and *dif* and has conventionally been used to map the origin and terminus of replication in diverse bacteria (24, 25). Curiously, the switch in the polarity of GC-skew occurs closer to *dif* than to any of the *Ter* sites, which points to a more convoluted relationship between chromosome replication and XerCD recombination (26).

**FIG 1:**
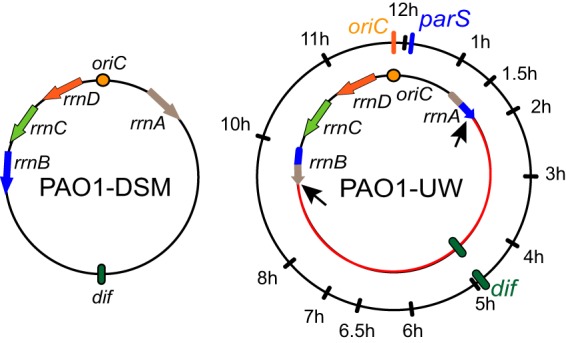
Genome organization of the two substrains of P. aeruginosa strain PAO1. In PAO1-UW, an inversion between two rRNA operons, *rrnA* and *rrnB*, resulted in an asymmetric location of *oriC* and *dif*. Location of the chromosomal tags used in this study is shown on the outer dial.

Pseudomonas aeruginosa is an opportunistic human pathogen responsible for serious nosocomial infection in newborns, patients with impaired immunity, and burn victims and is a leading cause of morbidity in cystic fibrosis patients (27, 28). Many P. aeruginosa genomes are asymmetrically organized with respect to *oriC* and *dif*. In particular, the reference P. aeruginosa strain, PAO1, exists in two main variations. The chromosome of one of them is nearly symmetric, whereas the left chromosomal arm of the other variant is 56% longer than the right arm (Fig. 1). This asymmetry is caused by a large inversion between two rRNA operons (29, 30). To conform to a previous classification, we refer to the symmetric variant as PAO1-DSM and to the asymmetric variant as PAO1-UW (29). Both strains were derived from the original isolate PAO, which has the same genomic arrangement as PAO1-DSM (29, 31). The exact instance when the inversion occurred is unknown (30). In PAO1-DSM, chromosome segregation proceeds sequentially from *parS* to *dif* (14, 32). Here, we investigated segregation in PAO1-UW.

We found that many details of chromosome segregation are conserved between the two PAO1 strains. In particular, the chromosomes in both strains are longitudinally organized, where *oriC* and *dif* are located at opposite poles of the cell. Likewise, segregation of chromosomal loci occurs in the middle of the cell, the site of replisome localization (32). Moreover, chromosome segregation occurs sequentially from *oriC* to *dif*, even though one of the arms is significantly longer than the other. As a further distinction from the symmetric strain, the longer arm of the PAO1-UW chromosome contains two large domains, which are extended along the cell but segregate together. Thus, the large-scale structure of the chromosome is adapted to its asymmetric subcellular layout. In contrast, the abundance of chromosomal markers declines symmetrically along the two arms, indicating that replication terminates opposite from *oriC* irrespective of the location of *dif*. Inspection of complete annotated genomes revealed that the asymmetric location of *oriC* and *dif* is common among bacteria. In all cases, GC-skew switched polarity in the vicinity of *dif* but not at the expected terminus of replication.

## RESULTS

### Segregation of *oriC* and *dif*.

**FIG 2:**
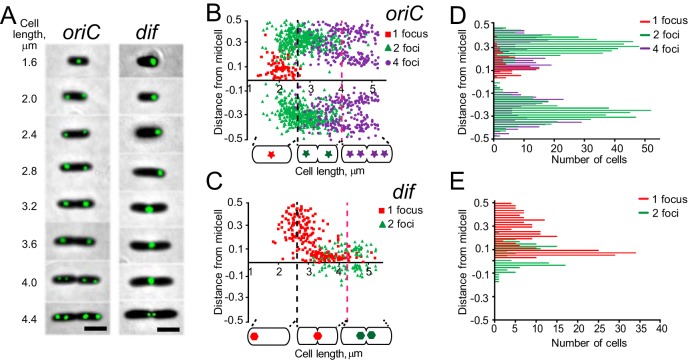
Segregation of *oriC* and *dif* in PAO1-UW. (A) Representative images of cells with tagged *oriC*- and *dif*-proximal loci. Scale bar is 2 μm. (B) Intracellular localization of *oriC*. Cells were binned according to the number of fluorescent foci per cell and quantified separately. Dashed lines mark midpoints of septum constriction and the second round of *oriC* segregation. The distance from midcell is given as a fraction of cell length. (C) Intracellular localization of the *dif*-proximal tag as a function of cell length. (D, E) Subcellular distribution of *oriC*- and *dif*-proximal tags.

To monitor chromosome segregation, cells with fluorescently labeled chromosomal loci were grown in M9 minimal medium containing 0.25% sodium citrate at 30°C. Under these conditions, the doubling time was 55 min, and at most, one new round of replication was initiated (but did not progress far) prior to cell division. Figure 2A shows representative cells with tagged *oriC*- and *dif*-proximal loci. In short cells, which correspond to early stages of the cell cycle, *oriC* was located in the middle of the cell and *dif* was located at a cell pole. As the cells grew, the *oriC* foci split and relocated to the quarters of the cell, whereas *dif* migrated toward the middle. Further growth of the cells was accompanied by another split of *oriC*, which was eventually followed by duplication of the *dif* locus. However, most cells divided prior to the second round of *oriC* segregation, giving rise to daughter cells with a single focus of *oriC*.

This pattern of separation can be also seen in Fig. 2B and C, where subcellular localization of all scored foci is plotted against the length of the cell. A single focus of *oriC* could be found only in cells shorter than 2.0 μm, whereas the second round of *oriC* segregation occurred in cells longer than 4.0 μm. Following duplication, *oriC* foci quickly migrated to their ultimate locations at the cell quarters (more precisely, 20% and 80% of the cell length; see also reference 32), resulting in distinct distributions centered around the midcell, cell quarters, or octet positions for single-, double-, or quadruple-focus cells, respectively (Fig. 2D). In contrast, the *dif* locus slowly relocated from the new pole toward the midcell and only after its duplication did its position produce a tight distribution (Fig. 2E).

**FIG 3:**
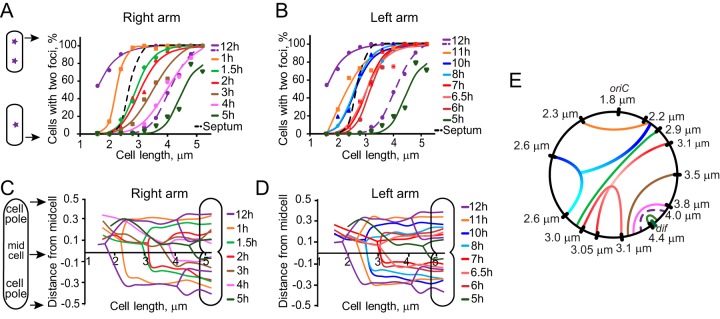
Segregation profiles of the tagged sites on the PAO1-UW chromosome. (A, B) Percentages of one- and two-focus cells as a function of cell size for sites located on the right (A) and left (B) chromosomal arms. The dashed line represents the second round of *oriC* segregation, i.e., the transition from two- to four-focus cells. Lines represent the best fit to a sigmoid function. Septum constriction is shown with a dashed black line. (C, D) Average location of each chromosomal locus at a given cell length. The lines are split at the midpoint of transition as determined in panels A and B. (E) Synchronicity of foci segregation on the two chromosomal arms. For each tagged locus, a match was found, by interpolation when needed, on the opposite arm that segregates at the same cell length.

To relate chromosome dynamics to cell growth and division, we next scored cells according to the presence of the septum. The existence of a septum was evaluated by visual inspection of phase-contrast images of the cells. The septum was considered to be formed when the undulation in the middle exceeded 10% of the average cell width. The midpoint for septum constriction was estimated as 2.7 μm (Fig. 3A). Thus, segregation of *oriC* preceded septum constriction, whereas relocation of *dif* toward the midcell occurred concurrently.

### P. aeruginosa chromosome segregates from *oriC* to *dif*.

Segregation patterns of all tagged chromosomal loci are summarized in Fig. 3. We first analyzed the cell length dependence of focus duplication. To this end, we binned the cells according to their lengths and separately counted those with one or two fluorescent foci. The fractions of cells having two foci were then plotted as a function of cell length for *tetO* tags located on the right (Fig. 3A) or left (Fig. 3B) chromosomal arms. We then quantified the formation of cells with four *oriC* foci, which was the only locus that underwent a second round of segregation.

The majority of newly born cells contained two *oriC* foci, indicating that segregation of *oriC* frequently occurs prior to cell division. This is consistent with the finding of the second round of *oriC* segregation in long cells, which occurs shortly before the segregation of *dif*. All other tested locations were present only in single copies in short cells and duplicated sequentially in accord with their genomic location for both the left and right chromosomal arms (Fig. 3A and B). The subcellular localization of the tagged loci before and after their separation is shown in panels C and D of Fig. 3. In all cases, foci duplication occurred close to the middle of the cell. This is consistent with the previously reported finding that the replisome is located at the midcell in PAO1 cells (32). Shortly after duplication, foci migrated away from the midcell (Fig. 3C and D).

Strikingly, the last segregated locus was found at 4.7 h in the vicinity of *dif*. The midpoint of *dif* segregation was at the cell length of 4.4 μm, which is significantly later than the 3.1-μm length found for the 6-h position or the 3.8-μm length found for the *tetO* tag at 4 h (Fig. 3E). Moreover, *dif* appeared as a single focus in 20% of the longest quantified cells, which further supports the notion of a delay in *dif* segregation. Thus, chromosome segregation in PAO1-UW proceeds unevenly along the two arms and terminates at *dif*.

### The P. aeruginosa PAO1-UW chromosome is longitudinally oriented.

We next determined whether or not the tagged chromosomal loci have a preferred location within the cell. We limited the analysis to two-focus cells where the difficulties in telling apart the new and old cell poles would not complicate the interpretation of the results. Indeed, the two cell poles appear identical in phase-contrast micrographs. As a result, foci located at the same distance from either the old or new pole appear to occupy the same position inside the cell.

**FIG 4:**
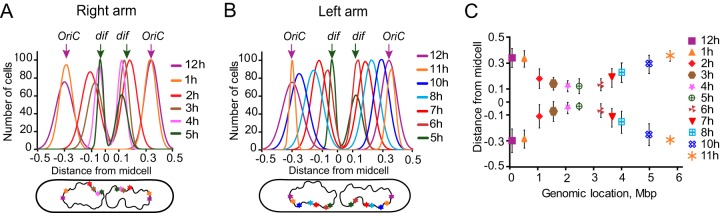
Longitudinal orientation of PAO1-UW chromosome. (A, B) Location of the indicated chromosomal loci in double-focus cells. The distribution of focal locations was approximated with a double Gaussian function (Fig. S2), and the best-fit Gaussian function was plotted as a function of subcellular localization for the sites on the right (A) and left (B) chromosomal arms. Genomic location of each site is illustrated on a cartoon below the graph. (C) Average (±SD) subcellular position of each locus plotted against its genomic location.

For each chromosomal tag, the distribution of focal locations was approximated as a double Gaussian distribution (Fig. S2), and the center of the Gaussian distribution was adopted as the average position of a given locus. We found that both chromosomal arms were largely stretched between *oriC* and *dif* (Fig. 4). The *oriC* locus was found at about 70% of the distance from the midcell to the cell pole, and *dif*, after its duplication, was located at about 15%. The other sites were located between these two extremes. The subcellular positions of the tagged loci paralleled their genomic locations (Fig. 4C). These results reveal that the chromosome in PAO1-UW cells is longitudinally oriented; following chromosome duplication, the two sister *oriC* sites occupy positions close to the cell poles, the two *dif* sites are in the middle of the cell, and the two chromosomal arms in each sister run in parallel between *oriC* and *dif*.

### The PAO1-UW chromosome segregates discontinuously along the arms.

**FIG 5:**
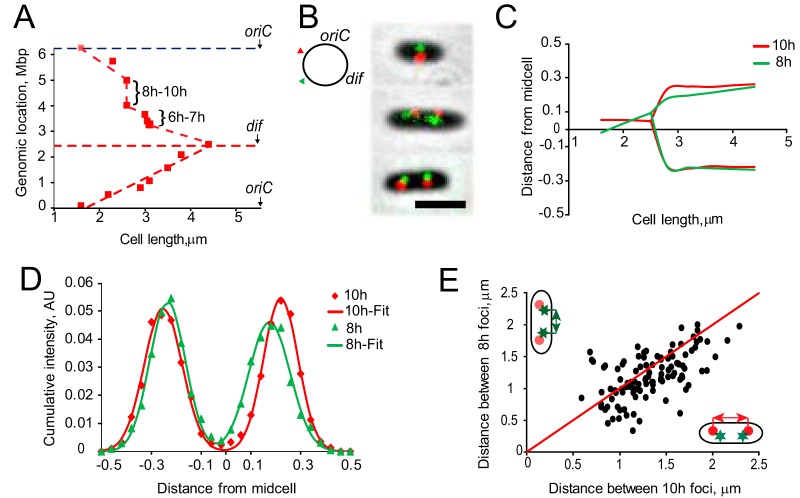
Discontinuity in segregation of PAO1-UW chromosome. (A) Genomic locations of foci that separate at a given cell length. Note two large domains that segregate simultaneously. (B) Overlaid phase and fluorescence images of representative cells labeled with mCherry-ParB^pMT1^ at 10 h and TetR-CFP at 8 h. Scale bar, 2 μm. (C) Simultaneous segregation of the 8-h and 10-h loci in the same cell. (D) Cumulative intensity distribution fit to a double Gaussian function of mCherry-tagged loci at 10 h and CFP-tagged loci at 8 h in the double-labeled cells (*n *>* *100). (E) Comparison of separations between the 8-h and 10-h sister foci. The diagonal line marks equal separations.

Despite their unequal lengths, the two arms of the PAO1-UW chromosome completed segregation at the same time. To gain insight into how the two segregation events are synchronized, we examined the timeline of chromosome segregation. To this end, we determined the cell length at which 50% of the population had two visible TetR-cyan fluorescent protein (CFP) foci for each tagged locus and then plotted these values against the genomic location of each tag (Fig. 5A). We found that chromosome segregation proceeds at a virtually constant rate along the short chromosomal arm but is discontinuous along the long arm. Two large segments of the chromosome, located between the 8-h and 10-h and between the 6-h and 7-h genomic coordinates, segregated as two distinct units at a constant cell length. This behavior appeared to apply to the entire stretch of the chromosome since the internal 6.5-h site segregated at the same cell length as its flanks at 6 h and 7 h (Fig. 3E and 5A). In this respect, these two domains, with the lengths of 1 Mbp and 0.5 Mbp, resemble the macrodomains previously described for E. coli (33–35).

To further characterize chromosome dynamics within the larger of the two domains, we tagged the 8-h and 10-h loci with two different tags, *tetO* and *parS^pMT1^* cassettes, and visualized the two tags in the same cells using TetR-CFP and ParB-mCherry, respectively (Fig. 5B). We found that indeed the two loci duplicated at the same time (Fig. 5C). Moreover, the distribution of focal locations was virtually the same for the tags at 8 h and 10 h, indicating that the sites are located close together most of the time (Fig. 5D). However, the average distance between the two 8-h loci was smaller than that for the 10-h sites (Fig. 5D). Inspection of individual cells further substantiated this observation. In 72% of the cells, the 8-h sister foci were closer together than the 10-h sister loci (*P = *10^−5^). Similar results were obtained when the two fluorescent tags were swapped at these locations (Fig. S3). Thus, although the two sites segregate at the same time, they occupy distinct positions within the cell.

### Chromosome replication terminates opposite from *oriC*.

**FIG 6:**
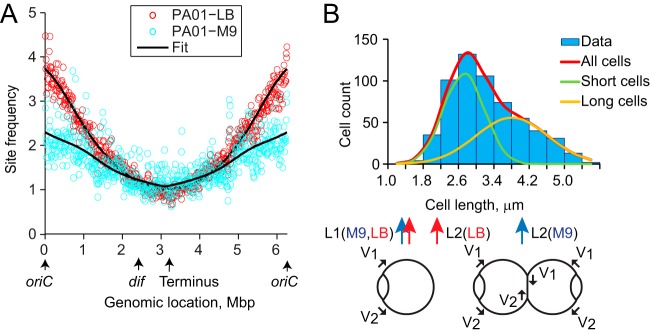
Replication proceeds symmetrically from *oriC*. (A) Copy numbers of various genomic sites for cells grown in LB and M9 medium, as indicated. The location of *oriC*, *dif*, and a site opposite from *oriC* (Terminus) are indicated below the graph. Lines show the fit to a model of replication fork outlined in panel B. (B) Cell size distribution in M9 medium fit to a double Gaussian function. The cartoon below the graph illustrates the model of replication fork progression (see Materials and Methods for details). Arrows indicate the best-fit cell length at which replication starts (*L*_1_ and *L*_2_ values in equation 1) for cells grown in M9 medium (blue) or LB (red).

We next evaluated the idea that replication of the PAO1-UW chromosome terminates at the *dif* locus. If true, this would offer a straightforward explanation as to why segregation terminates at *dif* despite asymmetry of the chromosome. To this end, we employed the genomics version of marker frequency analysis (36–38). Genomic DNA was extracted from both slow- and fast-growing PAO1-UW cells and then subjected to high-throughput sequencing. The relative abundance of each genomic marker was then plotted against its genomic coordinate (Fig. 6A) and fit to a model that postulates bidirectional replication that originates at *oriC* (Fig. 6B). This analysis builds on the expectation that the copy number of a marker doubles upon passage of the replication fork. As a result, the most- and least-abundant sites are expected to be found at the origin and terminus of replication, respectively.

We found a symmetric distribution of site abundancies with the highest copy number located at *oriC* and the lowest located opposite from it, at 3.2 Mbp (Fig. 6A). For comparison, the *dif* site is located at 2.4 Mbp. Thus, replication terminates away from *dif*. A similarly symmetric distribution was observed for cells grown in LB, albeit with a higher copy number of origin-proximal sites (Fig. 6A). Thus, replication of the PAO1-UW chromosome terminates opposite from *oriC* for both slow and fast growth conditions.

Fitting the data to a model revealed virtually identical replication rates for the clockwise and counterclockwise forks, 0.96 Mbp/μm and 0.98 Mbp/μm, respectively. In this model (see Materials and Methods for details), two populations of cells are expected, with two or four replication forks (Fig. 6B). Parameters of the model include the length of the cell at which a round of replication is initiated (*L*_1_ and *L*_2_) and the rates of the forks (*v*_1_ and *v*_2_). In addition, the cell length distribution measured under given conditions (Fig. 6B) is used to calculate the average expected frequency of each marker (see equation 2). Note that the rates of replication are expressed in the units of Mbp of DNA replicated per μm of cell growth (see equation 1). When the cell growth rate was taken into account, the best-fit rate of replication averaged to 0.94 kb/s.

For cells grown in M9 medium, replication was predicted to start only once per cell cycle at the average cell length of 4.0 μm (the value of *L*_2_ in Fig. 6B). As a result, the newly born cells were expected to have partially replicated chromosomes (since the best-fit *L*_1_ was shorter than the shortest cell) (Fig. 6B), in full accord with the results of microscopy studies (Fig. 2). In LB, the best-fit rates were 1.07 Mbp/μm and 1.02 Mbp/μm for the forks on the right and left chromosomal arms, respectively, but the second round of replication was predicted to occur sooner, in 2.0-μm cells (Fig. 6B), leading to a higher abundance of origin-proximal DNA (Fig. 6A).

### GC-skew switches polarity at *dif*, not opposite from *oriC*.

**FIG 7:**
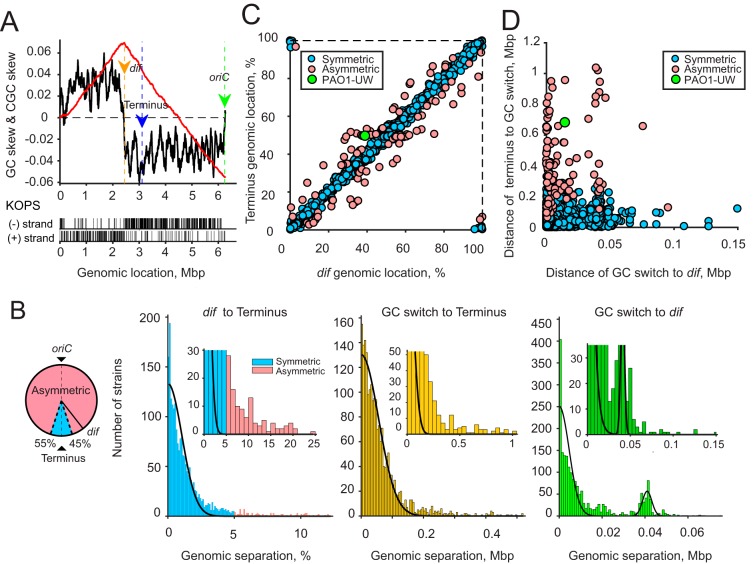
(A) Distribution of *dif* locations in the bacterial genomes GC-skew (black) and CGC-skew (red), with *oriC*, *dif*, and the replication terminus (top) and locations of KOPS sites (bottom) in the chromosome of PAO1-UW indicated. (B) Numbers of chromosomes with the indicated genomic separation between *dif* and the replication terminus (left histogram), the GC switch and the terminus (middle), and *dif* and the GC switch (right). The data were fit to a Gaussian distribution. Chromosomes where *dif* was located more than 5% away from the predicted terminus of replication were considered asymmetric. (C) Comparison of genomic locations, measured as a percentage of the chromosome length, of *dif* and the predicted terminus of replication in symmetric and asymmetric genomes. (D) Comparison of the proximities of the GC switch to *dif* and the expected terminus of replication.

Bacterial genomes display a notable nucleotide usage bias between the two DNA strands presumably due to a higher retention frequency of guanines rather than cytosines in the leading DNA strand (24, 25). This bias has been used to map the sites of initiation and termination of chromosome replication (39–41). In PAO1-UW, the positive DNA strand between *oriC* and 2.43 Mbp is enriched in guanines, whereas a higher frequency of cytosines is found in the rest of the positive strand of the chromosome (Fig. 7A). Notably, the switch in GC-skew polarity occurs within 15 kb from the *dif* site and 682 kb away from the replication termination site (Fig. 7A). Thus, GC-skew serves as a poor predictor of termination of chromosome replication in P. aeruginosa.

We next evaluated how common asymmetric genomes are in bacteria. As of 15 January 2018, the NCBI database contained 8,730 fully assembled genomes composed of 9,357 chromosomes, 4,055 of which contained a canonical *dif* sequence. To map *oriC* in these chromosomes, we examined their GC-skew profiles. Three thousand three hundred eighty-nine of these chromosomes consisted of two clearly defined domains, one each with a high and low guanine content. The remaining chromosomes contained multiple alternating GC-skew domains or highly asymmetric domains, suggestive of extensive chromosome rearrangements or, perhaps, multiple origins of replication. These genomes were excluded from further analysis.

On average, *dif* was found opposite from the predicted *oriC* gene. However, the distribution of *dif* locations was broad, with the width of the distribution spanning 1.5% of the chromosomal length (Fig. 7B). Chromosomes where *dif* was located more than 5% of the chromosomal length away from its expected location opposite from *oriC* were defined as asymmetric. This cutoff comprises 3.3 standard deviations of the Gaussian fit, with a >99% confidence interval (Fig. 7B). Out of 2,027 analyzed chromosomes, 113 were classified as asymmetric (Fig. 7B and C).

The switch in GC-skew polarity occurred much closer to *dif* than to the expected terminus of replication. The average distance from the GC-switch to *dif* was 13 kb as opposed to 71 kb for the terminus of replication (Fig. 7B). Notably, this pattern held true in both symmetric and asymmetric chromosomes (Fig. 7D), and the distribution of distances between the GC-switch and *dif* was indistinguishable between the two types of chromosomes (Fig. S4).

The GC-skew virtually mirrors the distribution of KOPS in PAO1-UW (Fig. 7A, bottom). Counting from *oriC* to *dif*, we found, respectively, 153 and 34 KOPS on the positive and negative strands of the short chromosome arm, and 271 and 57 KOPS on the negative and positive strands of the long arm. Given the high guanine content of KOPS, this bias contributes 595 and 1,070 excessive guanines to the overall GC-skew in the short and long arms, respectively. These numbers comprise only small fractions of the observed guanine excesses: 45,815 for the short arm and 81,869 for the long arm. Thus, the GC-skew and the KOPS bias appear to reflect different biological phenomena.

## DISCUSSION

We examined here segregation of a chromosome with unequal arms. This analysis revealed several features that remained masked in symmetric chromosomes. In particular, it became clear that chromosome replication and segregation are largely uncoupled from each other. Indeed, termination of replication and segregation of the PAO1-UW chromosome occurred at two different locations (Fig. 6). Based on marker frequency analysis, replication was predicted to terminate opposite from *oriC*. The abundance of sites on the two chromosomal arms was symmetric, suggesting that the replication forks move at virtually the same rate along the left and right arms. A similar conclusion has been previously reached from the analysis of the forks in E. coli, B. subtilis, and Caulobacter crescentus (36, 42–44). In contrast, chromosome segregation ended at the *dif* locus, which is separated by 0.8 Mbp from the predicted terminus of replication (Fig. 3E). Clearly, chromosome segregation was delayed at the *dif* locus, lagging behind its replication. Notably, an uncoupling of chromosome replication and recombination at *dif* has been previously observed in a laboratory strain with a large chromosomal inversion (23). Our data reveal that such strains are fairly widespread and do not have obvious fitness defects.

Notably, chromosome segregation was delayed not only at *dif* but at many other locations. For example, the 6 o’clock position, which coincides with the terminus of replication, segregated simultaneously with the 3 o’clock site found in the middle of the right chromosomal arms (Fig. 3E). Thus, the asymmetry of segregation propagated along both chromosomal arms. This asymmetry becomes especially clear in the comparison with the previous study of the symmetric strain PAO1-DSM, where segregation was proceeding continuously along both arms from *oriC* to *dif* (32). In this sense, the *dif* site appeared special and dictated the overall dynamics of chromosome segregation.

In further support of this view, we found that the global chromosome layout parallels its segregation order. Two sites, *oriC* and *dif*, were found at the opposite extremes of the chromosome, whereas the rest of the DNA was stretched between the two sites (Fig. 4). The average position of a site correlated with its genomic location (Fig. 4C). Likewise, segregation also proceeded from *oriC* to *dif*, sometimes discontinuously but otherwise in sync with the genomic location of the sites. Such a layout supports the view that chromosome segregation is driven by segregation of the replication origins, which is followed by a passive peeling apart of the emerging sister chromosomes. This order, however, is interrupted along the way (Fig. 3E), where local chromosome structure, or perhaps intertwining and tethering of the two arms, delays the progress of segregation along the shorter arms.

A striking feature of the PAO1-UW chromosome is the existence of two large domains, 1 Mbp and 0.5 Mbp long, which segregate discontinuously. DNA sites within these domains segregate at the same time but are located adjacent to each other (Fig. 5). The domains are reminiscent of the SNAP regions (45) or perhaps macrodomains described for E. coli, wherein DNA sites display unique dynamics and regulation consistent with tighter DNA packing within the macrodomains (33–35). Unsurprisingly, these domains are found in the longer arm of the PAO1-UW chromosome, which requires extra packing of DNA. Accordingly, no such domains were observed in the symmetric PAO1-DSM chromosomes (32). Thus, the mesoscale structure of the chromosome adjusts itself to accommodate its global layout. It is tempting to continue this line of reasoning and postulate that the local chromosome structure and, by extension, its activity are also affected by its global-scale subcellular organization.

Another intriguing implication of these findings is that the P. aeruginosa chromosome might be tethered to the cellular matrix at two locations, specifically, in the vicinity of *oriC* and *dif*, whereas the other interactions are circumstantial and have little bearing on cell division. The molecular organization of these tethers is yet to be determined. At the *oriC* end, the attachment is apparently mediated by the ParAB*S* system, which interacts with the chromosome and, sometimes, via extended tethers, the cell poles (8, 10, 46, 47). Which system is responsible for the attachment of the *dif* region is far less clear. The most likely known candidate for this role is the XerCD-FtsK system. Indeed, FtsK localizes to the septum and could, in principle, tether the chromosome to it (48, 49). This model, however, would need to explain how FtsK remains attached to *dif*-proximal DNA even after chromosome dimer resolution (Fig. 2C). In E. coli, the MatP protein is required for a delay in segregation of the Ter macrodomain (50). No such system has yet been found in P. aeruginosa.

This study offers new insights into the anatomy of GC-skew. Bacterial genomes display a virtually universal bias in the distribution of guanines and cytosines between the leading and lagging DNA strands. The origins of this phenomenon remain unknown. In a typical genome, GC-skew switches polarity in the vicinity of the origin of replication and *dif*. We show here that the same holds true even in asymmetric genomes. In such genomes, GC-skew does not correlate with the terminus of chromosome replication (Fig. 7). Clearly, an alternative mechanism for the link between GC-skew and *dif* needs to be proposed.

Such a mechanism could be an evolutionary pressure that acts to align the terminus of chromosome replication and the site of chromosome dimer resolution. Given that most of the chromosomes that carry a *dif* site are symmetric (Fig. 7B), such an arrangement appears to carry an evolutionary advantage. This mechanism, however, does not readily explain why the separation of *dif* from the GC-switch is severalfold smaller than its distance from the replication terminus (Fig. 7B). The evolution does not seem to have enough precision to ensure the observed average 13-kb separation between *dif* and the GC-switch. It seems more likely that at some point in time, *dif* was indeed located at the terminus of replication, perhaps at the time when it was acquired by the chromosome. In this view, most of the GC-skew would already have been introduced into bacterial genomes. The subsequent genetic drift would be sufficiently strong to separate the replication terminus and *dif* but not enough to reshuffle the GC-skew.

## MATERIALS AND METHODS

### Strains and plasmids.

Pseudomonas aeruginosa strains that were used in this study are listed in Table S1 in the supplemental material. PAO1 (ATCC 47085) was used as the wild-type strain. PCR analysis of the strain revealed an inversion between *rrnA* and *rrnB* genes (Fig. S1), which qualifies it as PAO1-UW (29). To tag defined locations in the PAO1-UW chromosome, approximately 500 bp of chromosomal segments at the desired locations were amplified and inserted between HindIII and KpnI sites of the pP30DFRT-*tetO*-0069 plasmid. This plasmid carries a cassette of approximately 140 *tetO* sequences (32, 51). The *tetO* cassette was then inserted into designated locations on the chromosome (see Tables S2 and S3 for the list of the used plasmids and primers) using homologous recombination (52).

10.1128/mBio.01088-18.1 FIG S1 Detection of the chromosomal inversion in PAO1 ATCC 47085. Download FIG S1, PDF file, 0.2 MB. Copyright © 2018 Bhowmik et al. 2018 Bhowmik et al. This content is distributed under the terms of the Creative Commons Attribution 4.0 International license.

10.1128/mBio.01088-18.2 FIG S2 Distribution of subcellular positions of the tagged loci. Download FIG S2, PDF file, 0.3 MB. Copyright © 2018 Bhowmik et al. 2018 Bhowmik et al. This content is distributed under the terms of the Creative Commons Attribution 4.0 International license.

10.1128/mBio.01088-18.3 FIG S3 Locations of mCherry-tagged 8-h and CFP-tagged 10-h loci. Download FIG S3, PDF file, 0.3 MB. Copyright © 2018 Bhowmik et al. 2018 Bhowmik et al. This content is distributed under the terms of the Creative Commons Attribution 4.0 International license.

10.1128/mBio.01088-18.4 FIG S4 Distribution of distances between the GC switch and *dif* for symmetric and asymmetric chromosomes. Download FIG S4, PDF file, 0.1 MB. Copyright © 2018 Bhowmik et al. 2018 Bhowmik et al. This content is distributed under the terms of the Creative Commons Attribution 4.0 International license.

10.1128/mBio.01088-18.5 TABLE S1 Strains used in this study. Download Table S1, PDF file, 0.1 MB. Copyright © 2018 Bhowmik et al. 2018 Bhowmik et al. This content is distributed under the terms of the Creative Commons Attribution 4.0 International license.

10.1128/mBio.01088-18.6 TABLE S2 Plasmids used in this study. Download Table S2, PDF file, 0.1 MB. Copyright © 2018 Bhowmik et al. 2018 Bhowmik et al. This content is distributed under the terms of the Creative Commons Attribution 4.0 International license.

10.1128/mBio.01088-18.7 TABLE S3 Oligonucleotides used in this study. Download Table S3, PDF file, 0.1 MB. Copyright © 2018 Bhowmik et al. 2018 Bhowmik et al. This content is distributed under the terms of the Creative Commons Attribution 4.0 International license.

Plasmid pPSV35Ap-TetR-CFP was constructed by removing yGFP-ParBT1 from pPSV35Ap-TetR-Cfp-yGfp-ParBT1 (32) using overlap extension PCR. The resulting plasmid carries an in-frame DNA segment encoding the tetracycline repressor protein (TetR) fused with the CFP at its C terminus. Expression of this chimera is controlled by an isopropyl-β-d-thiogalactopyranoside (IPTG)-inducible *lacUV5* promoter (32). In order to visualize the chromosomal tags, plasmid pPSV35Ap-TetR-CFP was introduced into the target cells using electroporation, and transformants were selected on LB agar plates.

To visualize two different segments of the chromosome simultaneously in live cells, the PAO1-UW chromosome was tagged with both a *parS^pMT1^* sequence and *tetO* arrays (32) and transformed with a pPSV35Ap-TetR-CFP-mCherry-ParBT1 plasmid. pPSV35Ap-TetR-CFP-mCherry-ParBT1 was constructed from pPSV35Ap-TetR-Cfp-yGfp-ParBT1 by replacing the GFP version of ParB^pMT1^ with that of the mCherry version.

### Bacterial growth and microscopy.

P. aeruginosa cells carrying chromosomal tags and the pPSV35Ap-TetR-CFP or pPSV35Ap-TetR-CFP-mCherry-ParBT1 plasmid were grown overnight at 37°C in M9 medium supplemented with 0.25% sodium citrate and a cocktail of trace ions (53). Whenever necessary, carbenicillin and gentamicin were added at 200 μg/ml and 30 μg/ml, respectively. The cells were then transferred into fresh M9 medium supplemented with 0.25% sodium citrate at an OD_600_ of 0.01 and incubated at 30°C. After 3 h of bacterial growth, expression of the fluorescent proteins was induced by the addition of 0.05 mM IPTG. Two hours after induction, the cells were collected at an OD_600_ of 0.1 and deposited onto an agarose pad (1% agarose in M9 medium supplemented with 0.25% sodium citrate) and observed using a fluorescence microscope (Olympus BX50 equipped with a BX-FLA mercury light source, a 100×, 1.43-numerical-aperture oil immersion objective). Fluorescent and phase-contrast images of cells were collected using a Spot Insight QE camera and analyzed using Nucleus (54).

### Replication profiling using marker frequency analysis.

Cells were grown at 30°C in M9 medium supplemented with 0.25% sodium citrate or in LB and harvested at an OD_600_ of 0.1 (M9 medium) or 0.6 (LB). The genomic DNA was then isolated using a GenElute bacterial genomic DNA kit (Sigma-Aldrich). The concentration and purity of isolated DNA were determined using a NanoDrop 2000c spectrophotometer (Thermo Scientific), and the cell size distribution was measured using phase-contrast microscopy. High-throughput sequencing of the P. aeruginosa genome was performed at the sequencing facility at OUHSC.

The relative abundancies of each DNA site determined using deep sequencing were binned and fit to a model of bidirectional replication using a home-written MATLAB script. The model postulates that each chromosomal arm contains two chromosomal arms, which advance at a constant rate. The copy number of a given site with a genomic location *x* was calculated as follows:

(1) $$n\left(x,\;L\right)=\left\{ \begin{array}{c} 4,x<v_{1}(L-L_{1})\;\text{or }(1-x)<v_{2}\left(L-L_{1}\right)\\ 2,x<v_{1}\left(L-L_{2}\right)\text{ or }(1-x)<v_{2}\left(L-L_{2}\right)\\ \text{ 1, otherwise} \end{array}\right.$$

where *v*_1_ and *v*_2_ are the velocities of the forks on the right and left arms, respectively, *L* is the cell length, and *L*_1_ and *L*_2_ are the parameters that specify at which cell length a given round of replication starts. The copy number of each site was then averaged over the entire cell population as follows:

(2) <n(x)>=∫n(x,L)p(L)dL

where *p*(*L*) is the probability of finding cells with a given length. This probability was determined by measuring the distribution of cell lengths at a given condition (Fig. 6B).

### Bioinformatic analysis.

The analysis of genome organization was carried out for 8,730 complete genomes comprised of 9,537 chromosomes, which were downloaded from the NCBI database (https://www.ncbi.nlm.nih.gov/genome/browse#!/prokaryotes/). A BLAST search among them, using the word-size of 11 nucleotides, identified 4,055 chromosomes, with a site homologous to the consensus *dif* sequence, AATTCGCATAATGTATATTATGTTAAAT (55). For these genomes, the GC-skew was calculated as (G-C)/(G+C), where G and C represent the number of guanines and cytosines, respectively, in a 1-kb window containing the site on the positive DNA strand. Three thousand four hundred twenty-one chromosomes contained two well-defined domains, one each with a positive and a negative GC-skew. Thirty-two of these chromosomes were disregarded because of their high asymmetry, when one of the domains occupied more than 75% of the entire chromosome. Out of these, 2,027 chromosomes contained a sequence with no more than 3 substitutions compared to the consensus *dif*. Six chromosomes contained two hits with fewer than 3 substitutions compared to the consensus sequence. Only one *dif* sequence with the highest similarity to the consensus was retained. For the remaining 2,027 chromosomes, the cumulative GC-skew, CGC, was computed, and the minimum and maximum on the CGC-skew profile, CGCmin and CGCmax, were determined. According to the previous analysis (56), the minimum on the CGC profile coincides with the location of *oriC*, and the maximum on the CGC profile marks the position of *dif* and the terminus of DNA replication.

## References

[1] McIntosh JR, Molodtsov MI, Ataullakhanov FI 2012 Biophysics of mitosis. Q Rev Biophys 45:147–207. doi:10.1017/S0033583512000017. 22321376 PMC4433171

[2] Nielsen HJ, Li Y, Youngren B, Hansen FG, Austin S 2006 Progressive segregation of the Escherichia coli chromosome. Mol Microbiol 61:383–393. doi:10.1111/j.1365-2958.2006.05245.x. 16771843

[3] Viollier PH, Thanbichler M, McGrath PT, West L, Meewan M, McAdams HH, Shapiro L 2004 Rapid and sequential movement of individual chromosomal loci to specific subcellular locations during bacterial DNA replication. Proc Natl Acad Sci U S A 101:9257–9262. doi:10.1073/pnas.0402606101. 15178755 PMC438963

[4] Wang X, Liu X, Possoz C, Sherratt DJ 2006 The two Escherichia coli chromosome arms locate to separate cell halves. Genes Dev 20:1727–1731. doi:10.1101/gad.388406. 16818605 PMC1522069

[5] Wang X, Llopis PM, Rudner DZ 2013 Organization and segregation of bacterial chromosomes. Nat Rev Genet 14:191–203. doi:10.1038/nrg3375. 23400100 PMC3869393

[6] Livny J, Yamaichi Y, Waldor MK 2007 Distribution of centromere-like parS sites in bacteria: insights from comparative genomics. J Bacteriol 189:8693–8703. doi:10.1128/JB.01239-07. 17905987 PMC2168934

[7] Godfrin-Estevenon A-M, Pasta F, Lane D 2002 The parAB gene products of Pseudomonas putida exhibit partition activity in both P. putida and Escherichia coli. Mol Microbiol 43:39–49. doi:10.1046/j.1365-2958.2002.02735.x. 11849535

[8] Ptacin JL, Lee SF, Garner EC, Toro E, Eckart M, Comolli LR, Moerner WE, Shapiro L 2010 A spindle-like apparatus guides bacterial chromosome segregation. Nat Cell Biol 12:791–798. doi:10.1038/ncb2083. 20657594 PMC3205914

[9] Murray H, Ferreira H, Errington J 2006 The bacterial chromosome segregation protein Spo0J spreads along DNA from parS nucleation sites. Mol Microbiol 61:1352–1361. doi:10.1111/j.1365-2958.2006.05316.x. 16925562

[10] Yamaichi Y, Fogel MA, McLeod SM, Hui MP, Waldor MK 2007 Distinct centromere-like parS sites on the two chromosomes of Vibrio spp. J Bacteriol 189:5314–5324. doi:10.1128/JB.00416-07. 17496089 PMC1951861

[11] Dubarry N, Pasta F, Lane D 2006 ParABS systems of the four replicons of Burkholderia cenocepacia: new chromosome centromeres confer partition specificity. J Bacteriol 188:1489–1496. doi:10.1128/JB.188.4.1489-1496.2006. 16452432 PMC1367244

[12] Breier AM, Grossman AD 2007 Whole-genome analysis of the chromosome partitioning and sporulation protein Spo0J (ParB) reveals spreading and origin-distal sites on the Bacillus subtilis chromosome. Mol Microbiol 64:703–718. doi:10.1111/j.1365-2958.2007.05690.x. 17462018

[13] Szardenings F, Guymer D, Gerdes K 2011 ParA ATPases can move and position DNA and subcellular structures. Curr Opin Microbiol 14:712–718. doi:10.1016/j.mib.2011.09.008. 21963112

[14] Lagage V, Boccard F, Vallet-Gely I 2016 Regional control of chromosome segregation in Pseudomonas aeruginosa. PLoS Genet 12:e1006428. doi:10.1371/journal.pgen.1006428. 27820816 PMC5098823

[15] May PFJ, Zawadzki P, Sherratt DJ, Kapanidis AN, Arciszewska LK 2015 Assembly, translocation, and activation of XerCD-dif recombination by FtsK translocase analyzed in real-time by FRET and two-color tethered fluorophore motion. Proc Natl Acad Sci U S A 112:E5133–E5141. doi:10.1073/pnas.1510814112. 26324908 PMC4577173

[16] Diagne CT, Salhi M, Crozat E, Salome L, Cornet F, Rousseau P, Tardin C 2014 TPM analyses reveal that FtsK contributes both to the assembly and the activation of the XerCD-dif recombination synapse. Nucleic Acids Res 42:1721–1732. doi:10.1093/nar/gkt1024. 24214995 PMC3919580

[17] Castillo F, Benmohamed A, Szatmari G 2017 Xer site specific recombination: double and single recombinase systems. Front Microbiol 8:453. doi:10.3389/fmicb.2017.00453. 28373867 PMC5357621

[18] Bigot S, Saleh OA, Lesterlin C, Pages C, El Karoui M, Dennis C, Grigoriev M, Allemand JF, Barre FX, Cornet F 2005 KOPS: DNA motifs that control E. coli chromosome segregation by orienting the FtsK translocase. EMBO J 24:3770–3780. doi:10.1038/sj.emboj.7600835. 16211009 PMC1276719

[19] Levy O, Ptacin JL, Pease PJ, Gore J, Eisen MB, Bustamante C, Cozzarelli NR 2005 Identification of oligonucleotide sequences that direct the movement of the Escherichia coli FtsK translocase. Proc Natl Acad Sci U S A 102:17618–17623. doi:10.1073/pnas.0508932102. 16301526 PMC1287487

[20] Hendrickson H, Lawrence JG 2006 Selection for chromosome architecture in bacteria. J Mol Evol 62:615–629. doi:10.1007/s00239-005-0192-2. 16612541

[21] Mulcair MD, Schaeffer PM, Oakley AJ, Cross HF, Neylon C, Hill TM, Dixon NE 2006 A molecular mousetrap determines polarity of termination of DNA replication in E. coli. Cell 125:1309–1319. doi:10.1016/j.cell.2006.04.040. 16814717

[22] Neylon C, Brown SE, Kralicek AV, Miles CS, Love CA, Dixon NE 2000 Interaction of the Escherichia coli replication terminator protein (Tus) with DNA: a model derived from DNA-binding studies of mutant proteins by surface plasmon resonance. Biochemistry 39:11989–11999. doi:10.1021/bi001174w. 11009613

[23] Cornet F, Louarn J, Patte J, Louarn JM 1996 Restriction of the activity of the recombination site dif to a small zone of the Escherichia coli chromosome. Genes Dev 10:1152–1161. doi:10.1101/gad.10.9.1152. 8654930

[24] Lobry JR 1996 Asymmetric substitution patterns in the two DNA strands of bacteria. Mol Biol Evol 13:660–665. doi:10.1093/oxfordjournals.molbev.a025626. 8676740

[25] McLean MJ, Wolfe KH, Devine KM 1998 Base composition skews, replication orientation, and gene orientation in 12 prokaryote genomes. J Mol Evol 47:691–696. doi:10.1007/PL00006428. 9847411

[26] Hendrickson H, Lawrence JG 2007 Mutational bias suggests that replication termination occurs near the dif site, not at Ter sites. Mol Microbiol 64:42–56. doi:10.1111/j.1365-2958.2007.05596.x. 17376071

[27] Lister PD, Wolter DJ, Hanson ND 2009 Antibacterial-resistant Pseudomonas aeruginosa: clinical impact and complex regulation of chromosomally encoded resistance mechanisms. Clin Microbiol Rev 22:582–610. doi:10.1128/CMR.00040-09. 19822890 PMC2772362

[28] Wagner VE, Iglewski BH 2008 P. aeruginosa biofilms in CF infection. Clin Rev Allergy Immunol 35:124–134. doi:10.1007/s12016-008-8079-9. 18509765

[29] Klockgether J, Munder A, Neugebauer J, Davenport CF, Stanke F, Larbig KD, Heeb S, Schöck U, Pohl TM, Wiehlmann L, Tümmler B 2010 Genome diversity of Pseudomonas aeruginosa PAO1 laboratory strains. J Bacteriol 192:1113–1121. doi:10.1128/JB.01515-09. 20023018 PMC2812968

[30] Stover CK, Pham XQ, Erwin AL, Mizoguchi SD, Warrener P, Hickey MJ, Brinkman FS, Hufnagle WO, Kowalik DJ, Lagrou M, Garber RL, Goltry L, Tolentino E, Westbrock-Wadman S, Yuan Y, Brody LL, Coulter SN, Folger KR, Kas A, Larbig K, Lim R, Smith K, Spencer D, Wong GK, Wu Z, Paulsen IT, Reizer J, Saier MH, Hancock RE, Lory S, Olson MV 2000 Complete genome sequence of Pseudomonas aeruginosa PAO1, an opportunistic pathogen. Nature 406:959–964. doi:10.1038/35023079. 10984043

[31] Holloway BW, Romling U, Tummler B 1994 Genomic mapping of Pseudomonas aeruginosa PAO. Microbiology 140:2907–2929. doi:10.1099/13500872-140-11-2907. 7812433

[32] Vallet-Gely I, Boccard F 2013 Chromosomal organization and segregation in Pseudomonas aeruginosa. PLoS Genet 9:e1003492. doi:10.1371/journal.pgen.1003492. 23658532 PMC3642087

[33] Valens M, Penaud S, Rossignol M, Cornet F, Boccard F 2004 Macrodomain organization of the Escherichia coli chromosome. EMBO J 23:4330–4341. doi:10.1038/sj.emboj.7600434. 15470498 PMC524398

[34] Niki H, Yamaichi Y, Hiraga S 2000 Dynamic organization of chromosomal DNA in Escherichia coli. Genes Dev 14:212–223. 10652275 PMC316355

[35] Lioy VS, Cournac A, Marbouty M, Duigou S, Mozziconacci J, Espeli O, Boccard F, Koszul R 2018 Multiscale structuring of the E. coli chromosome by nucleoid-associated and condensin proteins. Cell 172:771–783 e18. doi:10.1016/j.cell.2017.12.027. 29358050

[36] Khodursky AB, Peter BJ, Schmid MB, DeRisi J, Botstein D, Brown PO, Cozzarelli NR 2000 Analysis of topoisomerase function in bacterial replication fork movement: use of DNA microarrays. Proc Natl Acad Sci U S A 97:9419–9424. doi:10.1073/pnas.97.17.9419. 10944214 PMC16879

[37] Maduike NZ, Tehranchi AK, Wang JD, Kreuzer KN 2014 Replication of the Escherichia coli chromosome in RNase HI-deficient cells: multiple initiation regions and fork dynamics. Mol Microbiol 91:39–56. doi:10.1111/mmi.12440. 24164596 PMC3926323

[38] Skovgaard O, Bak M, Lobner-Olesen A, Tommerup N 2011 Genome-wide detection of chromosomal rearrangements, indels, and mutations in circular chromosomes by short read sequencing. Genome Res 21:1388–1393. doi:10.1101/gr.117416.110. 21555365 PMC3149504

[39] Sernova NV, Gelfand MS 2008 Identification of replication origins in prokaryotic genomes. Brief Bioinform 9:376–391. doi:10.1093/bib/bbn031. 18660512

[40] Frank AC, Lobry JR 2000 Oriloc: prediction of replication boundaries in unannotated bacterial chromosomes. Bioinformatics 16:560–561. doi:10.1093/bioinformatics/16.6.560. 10980154

[41] Kono N, Arakawa K, Tomita M 2012 Validation of bacterial replication termination models using simulation of genomic mutations. PLoS One 7:e34526. doi:10.1371/journal.pone.0034526. 22509315 PMC3317982

[42] Arias-Cartin R, Dobihal GS, Campos M, Surovtsev IV, Parry B, Jacobs-Wagner C 2017 Replication fork passage drives asymmetric dynamics of a critical nucleoid-associated protein in Caulobacter. EMBO J 36:301–318. doi:10.15252/embj.201695513. 28011580 PMC5286365

[43] Wang JD, Berkmen MB, Grossman AD 2007 Genome-wide coorientation of replication and transcription reduces adverse effects on replication in Bacillus subtilis. Proc Natl Acad Sci U S A 104:5608–5613. doi:10.1073/pnas.0608999104. 17372224 PMC1838449

[44] Kono N, Arakawa K, Sato M, Yoshikawa H, Tomita M, Itaya M 2014 Undesigned selection for replication termination of bacterial chromosomes. J Mol Biol 426:2918–2927. doi:10.1016/j.jmb.2014.06.005. 24946150

[45] Joshi MC, Bourniquel A, Fisher J, Ho BT, Magnan D, Kleckner N, Bates D 2011 Escherichia coli sister chromosome separation includes an abrupt global transition with concomitant release of late-splitting intersister snaps. Proc Natl Acad Sci U S A 108:2765–2770. doi:10.1073/pnas.1019593108. 21282646 PMC3041144

[46] Wu LJ, Errington J 2003 RacA and the Soj-Spo0J system combine to effect polar chromosome segregation in sporulating Bacillus subtilis. Mol Microbiol 49:1463–1475. doi:10.1046/j.1365-2958.2003.03643.x. 12950914

[47] Lin L, Osorio Valeriano M, Harms A, Sogaard-Andersen L, Thanbichler M 2017 Bactofilin-mediated organization of the ParABS chromosome segregation system in Myxococcus xanthus. Nat Commun 8:1817. doi:10.1038/s41467-017-02015-z. 29180656 PMC5703909

[48] Stouf M, Meile J-C, Cornet F 2013 FtsK actively segregates sister chromosomes in Escherichia coli. Proc Natl Acad Sci U S A 110:11157–11162. doi:10.1073/pnas.1304080110. 23781109 PMC3704039

[49] Bisicchia P, Steel B, Mariam Debela MH, Löwe J, Sherratt D 2013 The N-terminal membrane-spanning domain of the Escherichia coli DNA translocase FtsK hexamerizes at midcell. mBio 4:e00800–e00813. doi:10.1128/mBio.00800-13. 24302254 PMC3870252

[50] Mercier R, Petit M-A, Schbath S, Robin S, El Karoui M, Boccard F, Espéli O 2008 The MatP/matS site-specific system organizes the terminus region of the E. coli chromosome into a macrodomain. Cell 135:475–485. doi:10.1016/j.cell.2008.08.031. 18984159

[51] Lau IF, Filipe SR, Soballe B, Okstad OA, Barre FX, Sherratt DJ 2003 Spatial and temporal organization of replicating Escherichia coli chromosomes. Mol Microbiol 49:731–743. 12864855 10.1046/j.1365-2958.2003.03640.x

[52] Zhao H, Clevenger AL, Ritchey JW, Zgurskaya HI, Rybenkov VV 2016 Pseudomonas aeruginosa condensins support opposite differentiation states. J Bacteriol 198:2936–2944. doi:10.1128/JB.00448-16. 27528506 PMC5055598

[53] Schleheck D, Barraud N, Klebensberger J, Webb JS, McDougald D, Rice SA, Kjelleberg S 2009 Pseudomonas aeruginosa PAO1 preferentially grows as aggregates in liquid batch cultures and disperses upon starvation. PLoS One 4:e5513. doi:10.1371/journal.pone.0005513. 19436737 PMC2677461

[54] Wang Q, Mordukhova EA, Edwards AL, Rybenkov VV 2006 Chromosome condensation in the absence of the non-SMC subunits of MukBEF. J Bacteriol 188:4431–4441. doi:10.1128/JB.00313-06. 16740950 PMC1482961

[55] Carnoy C, Roten CA 2009 The dif/Xer recombination systems in proteobacteria. PLoS One 4:e6531. doi:10.1371/journal.pone.0006531. 19727445 PMC2731167

[56] Grigoriev A 1998 Analyzing genomes with cumulative skew diagrams. Nucleic Acids Res 26:2286–2290. doi:10.1093/nar/26.10.2286. 9580676 PMC147580

