# Supplementary material for: Segregation but Not Replication of the Pseudomonas aeruginosa Chromosome Terminates at Dif
Source: mBio. 2018 Oct 23;9(5):e01088-18. doi: 10.1128/mBio.01088-18 (PMC6199493; doi:10.1128/mBio.01088-18)
Supplement: FIG S1 [file mbo005184121sf1.pdf]

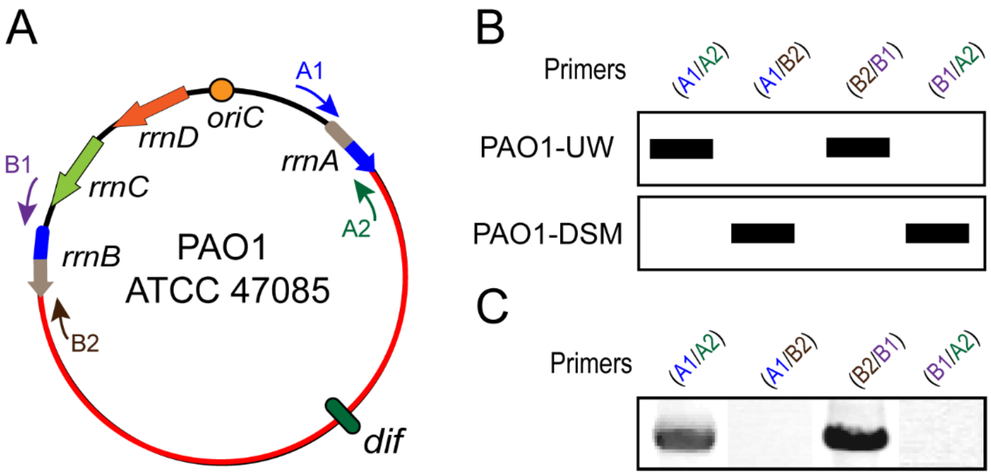

Figure S1. Detection of the chromosomal inversion in PAO1 ATCC 47085. (A) Location of *rRNA* operons in PAO1. Primers A1, A2, B1 and B2 match flanking regions of *rrnA* and *rrnB.* (B) Expected PCR amplicons from PAO1-UW and PAO1-DSM using various primer combinations. (C) PCR amplicons generated from the chromosome of PAO1 ATCC 47085.
