# Supplementary material for: Segregation but Not Replication of the Pseudomonas aeruginosa Chromosome Terminates at Dif
Source: mBio. 2018 Oct 23;9(5):e01088-18. doi: 10.1128/mBio.01088-18 (PMC6199493; doi:10.1128/mBio.01088-18)
Supplement: FIG S3 [file mbo005184121sf3.pdf]

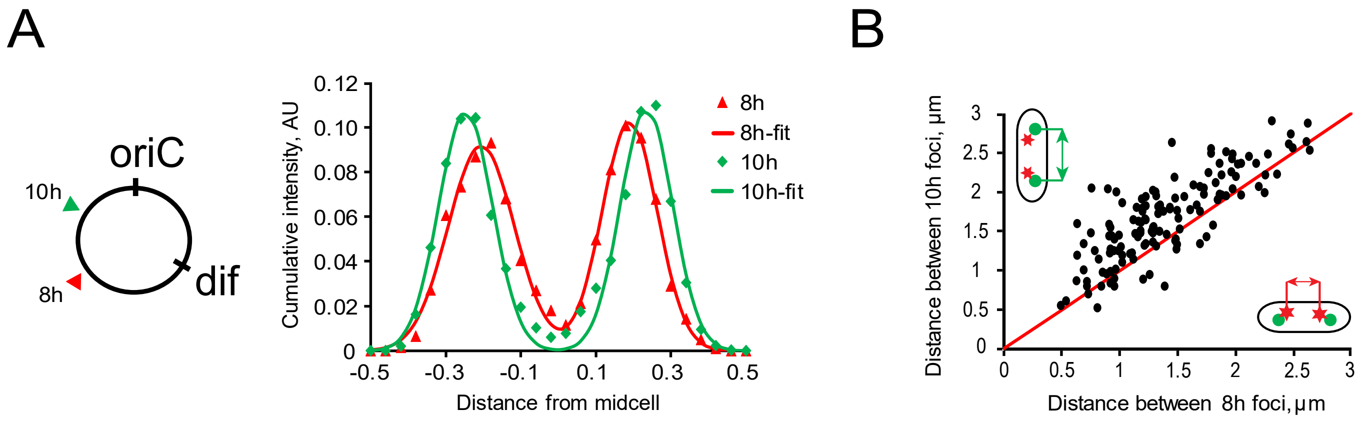

Figure S3. Location of mCherry-tagged 8h and CFP-tagged 10h loci. (A) Cumulative intensity distribution of the 8h and 10h loci in the double-labelled cells fit to a double-Gaussian distribution (*n* > 100). (B) A comparison of separations between the 8h and 10h sister foci in the double-labelled cells. The diagonal line marks equal separations.
