# Supplementary material for: Segregation but Not Replication of the Pseudomonas aeruginosa Chromosome Terminates at Dif
Source: mBio. 2018 Oct 23;9(5):e01088-18. doi: 10.1128/mBio.01088-18 (PMC6199493; doi:10.1128/mBio.01088-18)
Supplement: FIG S4 [file mbo005184121sf4.pdf]

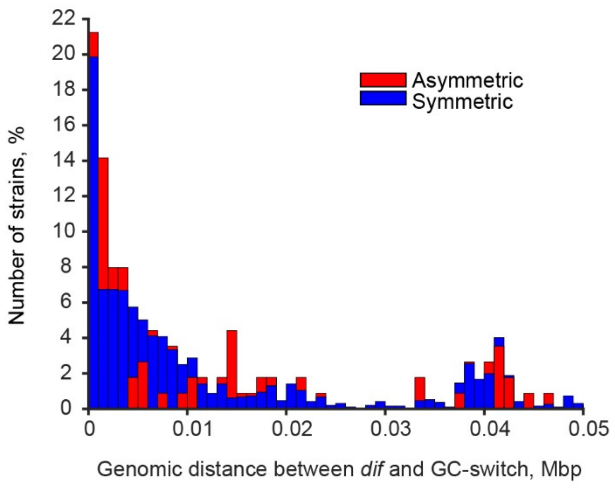

Figure S4. Distribution of distances between GC-switch and *dif* for symmetric and asymmetric chromosomes.
