# Supplementary material for: Segregation but Not Replication of the Pseudomonas aeruginosa Chromosome Terminates at Dif
Source: mBio. 2018 Oct 23;9(5):e01088-18. doi: 10.1128/mBio.01088-18 (PMC6199493; doi:10.1128/mBio.01088-18)
Supplement: TABLE S1 [file mbo005184121st1.pdf]

Table S1. Strains used in this study.

Strain	Relevant genotype or description	Source or reference
SM10 (λ pir)	*thi thr leu tonA lacY supE recA*::RP4-2-Tc::Mu Km λpir	(1)
DH5α	*supE44 ΔlacU169 hsdR17 recA1 endA1 gyrA96 thi-1 relA1*	Lab stock
PAO1	*lacI*$^{q+}$ delta(*lacZ*)M15^{+} *tetA*$^{+}$ *tetR*$^{+}$	ATCC 47085
OP342	PAO1 *tetO*-PA0069	This study
OP397	PAO1 *tetO*-PA0460	This study
OP405	PAO1 *tetO*-PA0716	This study
OP334	PAO1 *tetO*-PA0981	This study
OP411	PAO1 *tetO*-PA1436	This study
OP402	PAO1 *tetO*-PA1905	This study
OP335	PAO1 *tetO*-PA2258	This study
OP379	PAO1 *tetO*-PA2910	This study
OP428	PAO1 *tetO*-PA3035	This study
OP393	PAO1 *tetO*-PA3267	This study
OP341	PAO1 *tetO*-PA3573	This study
OP380	PAO1 *tetO*-PA4457	This study
OP419	PAO1 *tetO*-PA5099	This study
OP514	PAO1 *parS*^{pMT1}-PA4457-*tetO*-PA3753	This study

1. Hoang TT, Karkhoff-Schweizer RR, Kutchma AJ, Schweizer HP. 1998. A broad-host-range Flp-FRT recombination system for site-specific excision of chromosomally-located DNA sequences: application for isolation of unmarked Pseudomonas aeruginosa mutants. Gene 212:77-86.
