# Supplementary material for: Segregation but Not Replication of the Pseudomonas aeruginosa Chromosome Terminates at Dif
Source: mBio. 2018 Oct 23;9(5):e01088-18. doi: 10.1128/mBio.01088-18 (PMC6199493; doi:10.1128/mBio.01088-18)
Supplement: TABLE S2 [file mbo005184121st2.pdf]

Table S2. Plasmids used in this study. The exact location of each tag is shown in parenthesis; *dif* is located at 4.68h.

Plasmids	Description	Genomic location	Source or reference
pP30D-FRT-TetO-0069	For insertion of *tetO* repeats at 12h (0.16h)	PA0069	(1)
pP30D-FRT-TetO-0460	For insertion of *tetO* repeats at 1h (0.99h)	PA0460	This study
pP30D-FRT-TetO-0716	For insertion of *tetO* repeats at 1.5h (1.51h)	PA0716	This study
pP30D-FRT-TetO-0981	For insertion of *tetO* repeats at 2h (2.04h)	PA0981	(1)
pP30D-FRT-TetO-1436	For insertion of *tetO* repeats at 3h (2.99h)	PA1436	This study
pP30D-FRT-TetO-1905	For insertion of *tetO* repeats at 4h (3.98h)	PA1905	This study
pP30D-FRT-TetO-2258	For insertion of *tetO* repeats at 5h (4.76h)	PA2258	(1)
pP30D-FRT-TetO-2910	For insertion of *tetO* repeats at 6h (5.75h)	PA2910	This study
pP30D-FRT-TetO-3035	For insertion of *tetO* repeats at 6.5h (6.51h)	PA3035	This study
pP30D-FRT-TetO-3267	For insertion of *tetO* repeats at 7h (6.99h)	PA3267	This study
pP30D-FRT-TetO-3573	For insertion of *tetO* repeats at 8h (7.67h)	PA3573	(1)
pP30D-FRT-TetO-4457	For insertion of *tetO* repeats at 10h (9.6h)	PA4457	This study
pP30D-FRT-TetO-5099	For insertion of *tetO* repeats at 11h (10.99h)	PA5099	This study
pP30D-FRT-parST1-4457	For insertion of $parS^{pMT1}$ at 10h (9.6h)	PA4457	This study
pP30D-FRT-parST1-3573	For insertion of $parS^{pMT1}$ at 8h (7.67h)	PA3573	This study
pPSV35Ap-TetR-CFP	TetR-CFP expression plasmid		This study
pPSV35Ap-TetR-CFP-mCherry-ParBT1	TetR-CFP and mCherry-ParBT1 expression plasmid		This study

1. Vallet-Gely I, Boccard F. 2013. Chromosomal organization and segregation in Pseudomonas aeruginosa. PLoS genetics 9:e1003492.
