# Supplementary material for: Segregation but Not Replication of the Pseudomonas aeruginosa Chromosome Terminates at Dif
Source: mBio. 2018 Oct 23;9(5):e01088-18. doi: 10.1128/mBio.01088-18 (PMC6199493; doi:10.1128/mBio.01088-18)
Supplement: TABLE S3 [file mbo005184121st3.pdf]

Table S3. Oligonucleotides used in this study.

Plasmid	Primer sequence	Genomic location
pP30D-FRT-tetO-0460	GCACTGAAGCTTGGCAACGATTATTCGCAACTGG<br>GTACGTGGTACCACCCATCTCGTAGGGCGAATAACC	PA0460<br>(520737 bp)
pP30D-FRT-tetO-0716	GCACTGAAGCTTGGATTGGTAGAGGTCTCTGCAAAGG<br>GTACGTGGTACCAACATGAGAATTGCGATGACTCCC	PA0716<br>(786928 bp)
pP30D-FRT-tetO-0981	GCACTGAAGCTTGGTGTTGAGCCATATGGCGGATC<br>GTACGTGGTACCGCGGTTCATCTTTCCTCATAACTTGAGG	PA0981<br>(1062921 bp)
pP30D-FRT-tetO-1436	GCACTGAAGCTTGTGTACATCATCCTCGGCGTGC<br>GTACGTGGTACCCCGATCCTCTGTTCGCCTTCG	PA1436<br>(1563967 bp)
pP30D-FRT-tetO-1673	GCACTGAAGCTTGGAAATGCGCCATTCTGCTTCC<br>GTACGTGGTACCCCATCAGGGTTTCCTCGAAGGC	PA1673<br>(1824969 bp)
pP30D-FRT-tetO-1905	GCACTGAAGCTTCCTGGAGTCGGTGGAGTTCTGG<br>GTACGTGGTACCCGAGCTGATCATCCACCAGACC	PA1905<br>(2076311 bp)
pP30D-FRT-tetO-2910	GCACTGAAGCTTTTCGGCGCAGGAATGTCG<br>GTACGTGGTACCCCGGCTCGCTCCCGCCCA	PA2910<br>(2999192 bp)
pP30D-FRT-tetO-3035	GCACTGAAGCTTCCTTGAACCGCTCTGCGTCG<br>GTACGTGGTACCCAGCGCCGGGTAGTGGTCC	PA3035<br>(3398104 bp)
pP30D-FRT-tetO-3267	GCACTGAAGCTTCTACAGGATCTTCTCACTGCCG<br>GTACGTGGTACCGCACCGTAAAATGGTTCAACG	PA3267<br>(3653937 bp)
pP30D-FRT-tetO-4457	GCACTGAAGCTTGATGTTGTCGAAAATCGC<br>GTACGTGGTACCTGTTCAACGAATGTGGCG	PA4457<br>(4989305 bp)
pP30D-FRT-tetO-5099	GTACGTGGTACCGTTCCTGCTGACCTTCTTCACC<br>GCACTGAAGCTTCGATGATGTTCTCGACACAGG	PA5099<br>(5741524 bp)
pP30D-FRT-parST1-3573	CGCTATAAGCTTCTCGGCAGGTAGAAATCG<br>ACTGATGGTACCACGGGTTTTTTCAGAATC	PA3573<br>(4006145 bp)
A1	CGTTGCCTCTGTCCTTAATAAGG	721367 bp
A2	GCTGAAACTCTTGCGTGGACAG	727965 bp
B1	CGATTGAGAGCAGAAAGCAGCC	4787709 bp
B2	CTTTCTGTGGCTTGTGCAGATG	4795103 bp
